# Parenteral vaccination with recombinant EtpA glycoprotein impairs enterotoxigenic *E. coli* colonization

**DOI:** 10.1128/iai.00601-24

**Published:** 2025-05-01

**Authors:** Tim J. Vickers, David P. Buckley, Nazia Khatoon, Alaullah Sheikh, Bipul Setu, Zachary T. Berndsen, James M. Fleckenstein

**Affiliations:** 1 Division of Infectious Diseases, Department of Medicine, Washington University School of Medicine 12275, St. Louis, Missouri, USA; 2 Department of Biochemistry, University of Missouri Columbia, Columbia, Missouri, USA; 3 Infectious Diseases, Medicine Service, Veterans Affairs Saint Louis Health Care System, St. Louis, Missouri, USA; University of California San Diego School of Medicine, La Jolla, California, USA

**Keywords:** enterotoxigenic *Escherichia coli*, adhesins, immunization, heat-labile toxin, diarrhea

## Abstract

Enterotoxigenic *E. coli* (ETEC) causes hundreds of millions of cases of acute diarrheal illness in low- and middle-income regions, disproportionately in young children. To date, there is no licensed, broadly protective vaccine against these common but antigenically heterogeneous pathogens. One of the more highly conserved antigens of ETEC, EtpA, is an extracellular glycoprotein adhesin that preferentially binds to A blood group glycans on intestinal epithelia. EtpA contributes to increased severity of illness in A blood group individuals, elicits robust serologic and fecal antibody responses following infection, and has been associated with protection against subsequent infection. However, its utility as a protective antigen needs further examination. In the present studies, we examined whether parenteral vaccination with recombinant EtpA (rEtpA) could afford protection against intestinal colonization in a murine model of ETEC infection. Here, we demonstrate that intramuscular vaccination with rEtpA, adjuvanted with double mutant LT (dmLT), primes IgG predominant mucosal antibody responses to ETEC challenge. Notably, however, both antibody levels and avidity, as well as protection, were dependent on the vaccination schedule. Likewise, through electron microscopy polyclonal epitope mapping (EMPEM), we observed a different repertoire of epitopes targeted by antibodies after a more protracted vaccination schedule. Next, we explored the utility of IM immunization with alum-adjuvanted rEtpA. This elicited strong serologic and fecal IgG responses. Although accompanied by negligible IgA mucosal responses, EtpA alum-adjuvanted IM vaccination nevertheless protected against ETEC intestinal colonization. Collectively, these data suggest that EtpA could expand the portfolio of antigens targeted in ETEC subunit vaccine development.

## INTRODUCTION

Enterotoxigenic *Escherichia coli* (ETEC) is a heterogeneous, diarrheagenic pathogenic variant (pathovar) of *E. coli,* defined by the production of heat-labile and/or heat-stable enterotoxins (1). These pathogens are a leading cause of diarrheal illness in young children in low- and middle-income countries (LMICs) (2–4), as well as immunologically naïve travelers to areas where clean water and sanitation remain limited (5–12). These pathogens remain a significant cause of death (13) due to acute diarrheal illness among children less than five years of age (2).

Although the death rate from acute illness appears to have declined appreciably over the last several decades due to the implementation of oral rehydration therapy and other measures, infections with these pathogens continue largely unabated (14–16) and have been linked repeatedly to a number of non-diarrheal sequelae including micronutrient deficiencies (17), malnutrition, and growth impairment (3, 18–21). In addition, malnourished children are at substantially increased risk of death due to diarrhea caused by ETEC and other pathogens (22–24). The long-term morbidity associated with these pathogens is driven by subclinical intestinal damage, characterized by alterations in the absorptive architecture of the small intestine (25), a condition known as environmental enteropathy or enteric dysfunction (26–28).

Most ETEC vaccine development has focused on a group of pathovar-specific, plasmid-encoded antigens known as colonization factors (CFs). To date, at least 29 distinct antigens have been described (29). The ideal ETEC vaccine, oral or parenteral, would be broadly protective, prevent moderate to severe diarrhea among young children in LMICs during periods when they are most susceptible (6–24 months of age), and be easily integrated for co-administration with available vaccines (30).

The majority of enteric vaccines developed to date, including the most advanced ETEC vaccine to enter clinical trials (31), have relied on oral delivery (32). Notably, however, children with enteropathy tend to respond poorly to oral vaccines, while their responses to parenterally delivered vaccines remain unimpaired (33, 34). Parenteral subunit vaccination has been shown to induce effective mucosal immune responses (33, 35, 36) and could potentially overcome limitations inherent to oral vaccination in LMICs. The development of fimbrial tip adhesin molecules representing some of the more common CF antigens has provided a precedent for the pursuit of a parenteral subunit vaccine strategy (37, 38). However, given the heterogeneity of these antigens, it appears likely that other molecules will need to be targeted to achieve broad protection.

One possible candidate antigen, EtpA, is a high molecular weight extracellular ETEC glycoprotein adhesin (39, 40) secreted by a wide variety of enterotoxigenic *E. coli*, including some strains without a recognized colonization factor (41). Molecular epidemiology studies suggest that EtpA-expressing strains are widely geographically distributed (42), and birth cohort studies indicate that this molecule contributes to symptomatic illness in young children (43). EtpA is recognized during natural and experimental (44, 45) human ETEC infections, and prior antibodies to EtpA appear to be associated with a decreased risk for subsequent infections (43).

EtpA, like many bacterial adhesins (46), is a lectin. It specifically facilitates ETEC interactions with A blood group glycans on intestinal epithelial cells to promote bacterial adhesion and toxin delivery (47). Intriguingly, both children (18) and adult human volunteers (47) expressing A blood group antigens appear to have an increased risk of symptomatic ETEC infection. Recent structure–function studies of EtpA, along with the determination of the molecular structure by cryo-EM, combined with epitope mapping of anti-EtpA monoclonal antibodies, have demonstrated that a series of repeat modules comprising the C-terminal region of the molecule direct critical interactions with A blood group glycans to mediate enterocyte adhesion (48).

Elucidation of the nature of these EtpA interactions with the host could be relevant to optimizing its use as a candidate subunit vaccine antigen. To date, recombinant EtpA vaccination via intranasal (49–51), oral, and sublingual (52) routes has been associated with protection against intestinal colonization in a murine model. To explore the utility of EtpA, as a parenterally administered antigen subunit, we vaccinated mice intramuscularly with EtpA and examined serologic and mucosal responses to vaccination and protection against infection with ETEC. Here, we demonstrate that EtpA is significantly immunogenic when delivered via IM vaccination, although antibody maturation and protection are dependent on the timing of immunizations.

## MATERIALS AND METHODS

### Recombinant EtpA production

Recombinant EtpA glycoprotein was produced as previously described (53). Briefly, Top10 (pJL017, pJL030) (Table 1) was grown from frozen glycerol stocks maintained at −80°C overnight in 75 mL terrific broth containing carbenicillin (100 µg/mL), chloramphenicol (25 µg/mL), and 0.2% glucose, at 37°C, 225 RPM. Overnight growth was then diluted the following morning 1:100 in 2-L flasks containing 500 mL of fresh media. After growth to an OD_600_ of ~0.6, expression was induced with arabinose at a final concentration of 0.0002% for 4.5 hours. The supernatant was recovered by centrifugation at 11,000 × *g* for 10 minutes, filtered through a 0.2-µm filter, and then concentrated by tangential flow (Pellicon 2 Biomax, 100 kDa MWCO) to ~100 mL. rEtpA was then captured on two 5 mL metal affinity chromatography columns (HisTrap HP, Cytiva Life Sciences) and washed with five column volumes of binding buffer (50 mM PO_4_, 300 mM NaCl, pH 7.5). Endotoxin depletion was integrated into subsequent column washes using ~20 column volumes of freshly prepared 0.1% (v/v) Triton X-114 (Sigma) in cold PBS (54). The column was then washed with binding buffer (~10 column volumes) until A_280_ was returned to baseline. Recombinant polyhistidine-tagged EtpA was eluted over a gradient of elution buffer (50 mM PO_4_, 300 mM NaCl, 1 M imidazole, pH 7.5). EtpA-containing fractions were identified by SDS-PAGE, pooled, and dialyzed against 10 mM MES, 100 mM NaCl, 1 mM EDTA, pH 6, and concentrated to a final concentration of ~1 mg/mL. Endotoxin levels (EU) were determined spectrophotometrically (Endosafe-nexgen-PTS, Charles River). Lots used in vaccination were determined to have endotoxin levels <50 EU/mL.

**TABLE 1:** Bacterial strains and plasmids used in these studies^*a*^

Strain	Description	Source/reference
H10407	Wild-type ETEC strain, LT/STh/STp; EtpA	(55)
Top10	F^-^ *mcrA* ∆(*mrr-hsdRMS-mcrBC*) ϕ80*lacZ∆*M15 ∆*lacX74 recA1 araD139* ∆(*ara* leu)7697 *galUgalK rpsL* (Str^R^) *endA1 nupG*	Invitrogen
jf876	H10407 *lacZYA*::Km^R^	(56)
jf1696	Top10 (pJL017, pJL030) Amp^R^, Cm^R^	(53)

Plasmid	Description	Addgene/reference
pJL030	pACYC184-based *etpC* transglycosylase expression plasmid, Cm^R^	53533/(40)
pJL017	pBAD/Myc-HisA-based etpBA expression plasmid, Amp^R^	53532/(49)

^*a*^ Str^R^, Cm^R^, Amp^R^, Km^R^: streptomycin, chloramphenicol, ampicillin, and kanamycin resistance, respectively.

### EtpA biotinylation

rEtpA in 10 mM MES (2-(N-Morpholino)ethanesulfonic acid hydrate, Sigma M8250), 100 mM NaCl, pH 6.0, was reacted at room temperature for 30 minutes with a tenfold molar excess of NHS-LC-LC-biotin (Thermo Fisher Scientific 21338) following the manufacturer’s directions. The reaction was then quenched with 100 mM Tris (pH 8.0), followed by dialysis in MES buffer to remove free biotin. Biotin incorporation was confirmed by immunoblotting with streptavidin-HRP.

### Enzyme-linked immunosorbent assay (ELISA)

#### EtpA ELISA

rEtpA (1 µg/mL) in carbonate buffer (pH 9.6) was used to coat wells (100 µL/well) overnight at 4°C and then washed three times with 200 µL of PBS-0.05% Tween-20 (PBS-T). Plates were blocked by incubation with 200 µL/well of 1% BSA in PBS-T for 1 hour at 37°C. Primary sera were diluted in PBS-T-BSA as indicated, and 100 µL was added to respective wells for 1 hour at 37°C. Wells were washed five times with PBS-T, and then horse anti-mouse IgG-HRP conjugate secondary antibody (Cell Signaling 7076) (Table 2) was added in PBS-T-BSA (100 µL/well) and incubated at 37°C for 1 hour. Wells were again washed five times with PBS-T and detected using freshly prepared TMB peroxidase substrate (3,5,3′,5′-tetramethylbenzidine, Seracare 5120–0053) and then read kinetically (57) at 650 nm (Eon, Biotek).

**TABLE 2:** Antibodies used in these studies^*a*^

Antibody	Description	Source	Catalog no.	RRID
Anti-A blood group	Mouse monoclonal IgM (mAb Z2a) raised vs. human A blood group antigen	Santa Cruz Biotechnology	69951	AB_1119619
Anti-mouse IgG	Polyclonal horse anti-mouse IgG (H&L) affinity-purified	Cell Signaling	7076	AB_330924
Anti-mouse	Alexa Fluor 647 conjugated goat anti-mouse IgM heavy chain	Molecular Probes	A21238	AB_2535807
Anti-O78	Rabbit anti-O78 polyclonal antisera	Penn State *E. coli* Reference Center	–	–
Anti-rabbit	Goat anti-rabbit IgG (H&L). Alexa Fluor 488 conjugate	Invitrogen	A32731	AB_2633280
Anti-rabbit	Goat anti-rabbit IgG (H&L). Alexa Fluor 594 conjugate	Invitrogen	A11012	AB_2534079

^*a*^ "–" indicates there are no catalog numbers for these sera and no available RRID numbers.

#### IgG avidity assay

ELISA plates were coated with rEtpA (1.0 µg/mL) in carbonate buffer (pH 9.6) overnight at 4°C. Plates were then washed six times with PBS-0.05% Tween-20 and blocked with 1% BSA in PBS-0.05% Tween-20 for 1 hour at 37°C. Sera, diluted 1:100,000 in blocking buffer, were incubated for 1 hour at 37°C and then washed with PBS. Half of the plate was incubated for an additional 5 minutes in PBS, while the other half was treated for 5 minutes with 8M urea in PBS. After washing, affinity-purified horse anti-mouse IgG conjugated to HRP (Cell Signaling 7076), diluted 1:5,000 in PBS-T-BSA, was added and incubated for 1 hour at 37°C. Plates were then washed with PBS and developed using freshly prepared TMB substrate. Antibody avidity was calculated as the ratio of kinetic ELISA responses in urea-treated wells relative to corresponding untreated wells (58).

#### Blood group A-EtpA binding ELISA

To examine EtpA binding to human A blood group (bgA), BSA-conjugated blood group A (MO Bi Tec Dextra NGP6305) was dissolved to a final concentration of 0.5 mg/mL in PBS containing 0.02% (w/v) azide and stored at 4°C immediately prior to use. The bgA-BSA conjugate working solution was then prepared in carbonate buffer (pH 9.6) at a final concentration of 1 µg/mL. ELISA strips (Corning 2580) were incubated overnight (100 µL/well) at 4°C, then washed three times with 200 µL of PBS containing 0.02% Tween-20 (PBS-T), and blocked with 100 µL of 1% BSA in PBS-T at 37°C for 1 hour. A 100 µL of biotinylated EtpA-biotin (10 µg/mL in PBS-T-1% BSA) was added per well, incubated for 2 hours at room temperature, and then washed five times with PBS-T. Wells were then incubated for 1 hour with avidin-HRP conjugate (BioRad 1706528, diluted 1:10,000 in 1% BSA in PBS-T) at room temperature and then washed again four times with PBS-T. Finally, wells were developed with freshly prepared, room-temperature HRP substrate (TMB-(3,5,3′,5′-tetramethylbenzidine)−2 component reagent (Seracare 5120–0053) and read kinetically at 650 nm.

### Intramuscular vaccination

Mice were vaccinated intramuscularly (IM) with 10 µg of rEtpA and 1 µg of double (R192G/L211A) (59) mutant LT (dmLT) in a final volume of 50 µL. Control mice were vaccinated with 1 µg of dmLT alone or an equal volume of PBS. Mice were vaccinated in two immunization schedules: days 0, 14, and 28, or days 0, 21, and 42. Alternatively, mice were vaccinated on days 0, 21, and 42 with either aluminum phosphate adjuvant alone (Adju-Phos vac-phos-250, InvivoGen, San Diego, CA, USA), or 10 µg of rEtpA adjuvanted with Adju-Phos in a 1:1 volume-to-volume ratio.

### Murine small intestinal colonization

Colonization studies were carried out in streptomycin-treated adult CD-1 mice, as previously described (52, 60). Strain jf876 was grown overnight in kanamycin (25 µg/mL) (Table 1) in 2 mL of LB at 37°C, 225 rpm, diluted in fresh media the morning of challenge, and grown to OD600 of ~0.3. Mice were challenged by gavage with ~10^5^ colony-forming units. On the day after the challenge, small intestinal (ileum) segments of ~3 cm length were isolated, lysed in saponin (5%) for 10 minutes, and serial dilutions plated onto Luria agar plates containing kanamycin. All experiments were conducted with the approval of the Animal Care and Use Committee at Washington University School of Medicine.

### ETEC adhesion to blood group A intestinal epithelia

HT-29 cells (ATCC HTB-38), which express A blood group glycans (47), were propagated as previously described in McCoy’s 5A medium (Gibco, Life Technologies) supplemented with 10% bovine serum albumin. Cells were grown to confluence in 96-well plates at 37°C with 5% CO_2_. Adhesion assays were performed as previously described (47) using mid-log phase bacterial cultures. After 30 minutes, monolayers were washed three times with pre-warmed media and then treated with 0.1% Triton X-100 in PBS for 5 minutes. Dilutions of the resulting lysates were plated onto Luria agar, and bacterial adherence was expressed as a percentage of the original inoculum recovered. Alternatively, adherent bacteria were identified using confocal microscopy.

### Confocal laser scanning microscopy

HT-29 cells seeded onto pre-treated poly-L-lysine glass coverslips in 24-well plates were incubated and grown as above at 5% CO_2_ with 37°C until confluent. ETEC H10407 was added at a multiplicity of infection of ~1:100 and incubated for ~30 minutes prior to fixation. Plasma membranes were stained with CellMask Deep Red (Thermo Fisher Scientific, C10046) (1:2,000) and nuclei with DAPI (1:6,000). A blood group (BgA) was detected using mouse monoclonal antibody Z2A (Santa Cruz sc-69951) against the human A blood group antigen, followed by Alexa Fluor 647-conjugated goat anti-mouse IgM heavy chain (Molecular Probes, A21238). Confocal images were acquired using a Nikon Eclipse Ti2 inverted microscope. ETEC H10407 (serotype O78) was imaged using polyclonal antisera (rabbit) supplied by the Penn State *E. coli* Reference Center, followed by cross-absorbed goat anti-rabbit IgG (H&L) conjugated to either Alexa Fluor 488 or 594 fluorophores (Invitrogen).

### Electron microscopy

#### Negative stain electron microscopy polyclonal epitope mapping (nsEMPEM) sample preparation

Samples of ~1 mg/mL IgG purified from sera of mice vaccinated with rEtpA/dmLT at two- or three-week intervals were incubated with 0.5 mL of immobilized papain resin (Thermo Fisher Scientific) for 6 hours at 37°C to liberate Fab from Fc regions of the antibodies. The digestion mixtures of two- and three-week polyclonal samples were separated from the papain resin by centrifugation at 4,698 × *g* for 10 minutes. Size-exclusion chromatography (SEC) was performed on each isolated digestion mixture using a Superdex 200 Increase 10/300 GL column (Cytiva), and fractions from the Fab/Fc-containing peak were pooled. Pooled two- and three-week samples were buffer-exchanged in parallel into the rEtpA suspension buffer (10 mM MES, 100 mM NaCl, pH 6.0) and subsequently concentrated to ~0.1 mg/mL using 10 kDa cutoff Amicon Ultra centrifugal filter units (Sigma-Aldrich). Fab-rEtpA complexes were formed by incubating a 6× molar excess of each purified Fab/Fc two- and three-week samples with 15 µg rEtpA to a final volume of 300 µL and stored at 4°C for 36 hours. SEC was repeated on the incubated samples to separate rEtpA-Fab complexes from free rEtpA/Fab/Fc.

#### Negative stain electron microscopy (nsEM) data acquisition

Freshly purified two- and three-week rEtpA-Fab complexes were concentrated to ~0.05 mg/mL. For each sample, 3 µL was pipetted onto glow-discharged, carbon-coated 300-mesh Cu grids and immediately blotted with filter paper. After repeated pipetting and blotting (×2), grids were negatively stained by incubating 3 µL of 2% (w/v) uranyl acetate for 30 seconds. Data were collected on the 120 keV JEOL JEM-1400Plus electron microscope at the Washington University Center for Cellular Imaging (WUCCI). A magnification of ×30,000 was used throughout data collection, with a nominal pixel size of 3.54 Å. Micrographs were obtained manually using an AMT XR111 high-speed 4k × 2k pixel phosphor-scintillated 12-bit CCD camera. CryoSPARC v4.6.2 (61) was used for all data processing.

#### nsEMPEM data processing

Elliptical blob-picking and subsequent pick filtering were performed on 323 micrographs for the two-week rEtpA-Fab dataset, resulting in a final set of 2,574 rEtpA-Fab particles (~9 particles/micrograph) after several rounds of two-dimensional (2D) classification followed by subset selection. Ten final, representative 2D classes (of 1,575 particles) were obtained for the two-week dataset after 2D class rebalancing, subset selection, and additional 2D classification. Manual picking of particles from 934 micrographs for the three-week rEtpA-Fab dataset was performed, and 3,439 rEtpA-Fab particles (~4 particles/micrograph) were obtained after several rounds of 2D classification followed by subset selection. 2D classes of manually curated particles from the three-week rEtpA-Fab dataset were used to perform a secondary template-based particle picking, which was combined with the original manually picked particles through the “Remove Duplicates” job following several more rounds of 2D classification and subset selection (resulting in a final total particle count of 4,956). Twenty final, representative 2D classes were split into two sets of ten 2D classes: one that highlights multiple Fabs bound to EtpA (1,338 particles) and the other highlights single Fabs bound to EtpA (1,527 particles) through 2D class rebalancing and subsequent rounds of 2D classification.

## RESULTS

### Parenteral vaccination primes mucosal responses to EtpA

**Fig 1:**
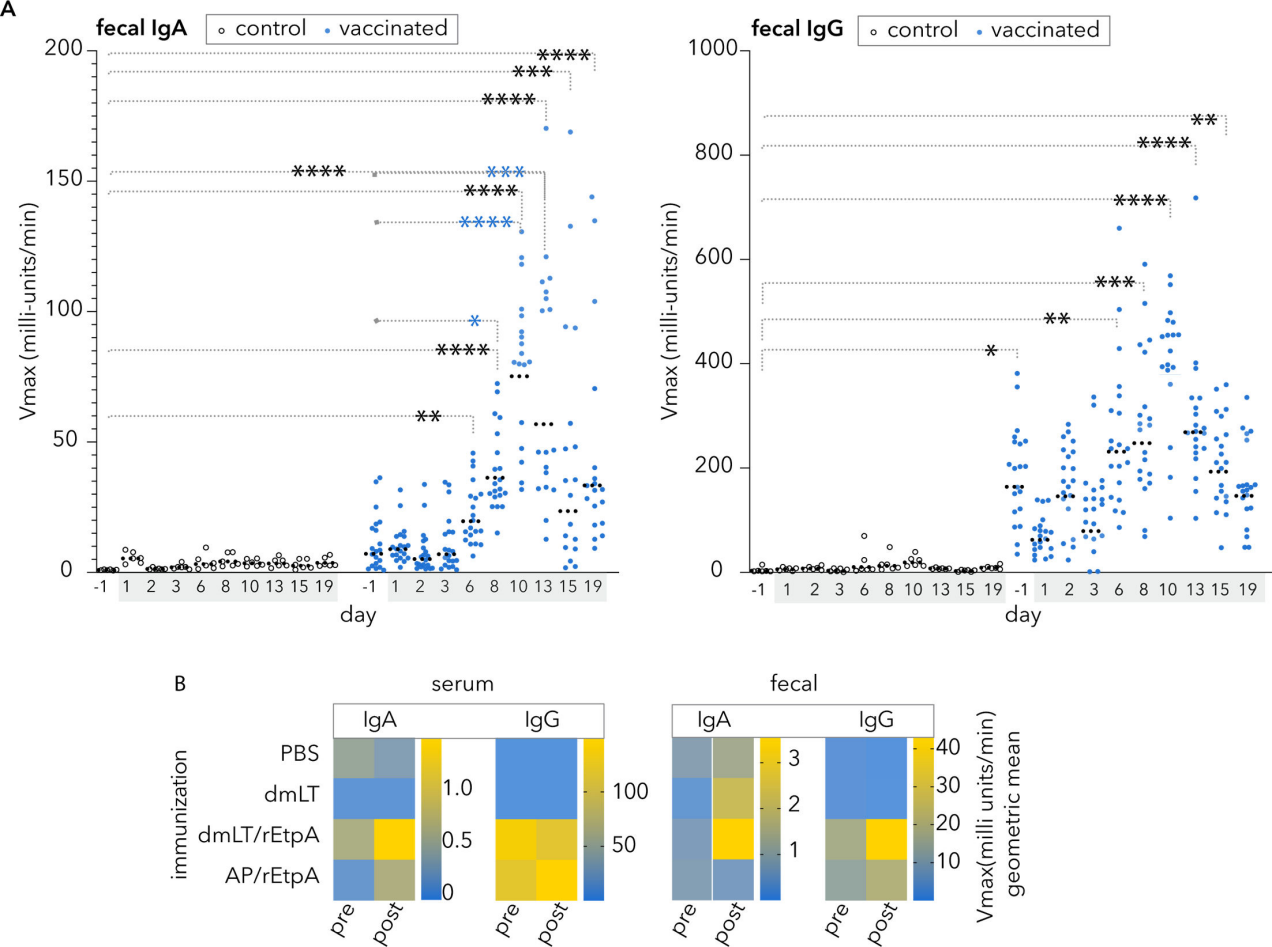
Parenteral vaccination with recombinant EtpA (rEtpA) adjuvanted with dmLT elicits mucosal antibody responses and enhances antigen responses following infection. (**A**) Graphs depict anti-EtpA fecal antibody responses by kinetic ELISA for sham-vaccinated (PBS, open circles) controls (*n* = 6), and mice vaccinated with rEtpA adjuvanted with dmLT (blue symbols, *n* = 20), before (day −1) and after (shaded days) infection with ETEC H10407 (1.5 × 10^7^ cfu). Fecal resuspensions were tested at 1:4 for both isotypes. Comparisons by Kruskal–Wallis testing: *<0.05, **<0.01, ***<0.001, ****<0.0001. Significant comparisons to PBS d-1 controls and vaccinated d-1 are shown in black and blue, respectively. Dashed horizontal lines represent geometric means. (**B**) Heatmaps summarize anti-EtpA kinetic ELISA data (geometric mean values) following vaccination of groups of mice (*n* = 5) with PBS (sham), dmLT adjuvant alone, rEtpA adjuvanted with dmLT (dmLT/rEtpA), or rEtpA adjuvanted with AdjuPhos (AP). Sera were tested at 1:500 dilution (IgA) and 1:100,000 (IgG). Fecal samples were assessed at a 1:10 dilution. Pre- and post-challenge samples were obtained on day −1 prior to and day 8 following infection with ETEC H10407 (2 × 10^5^ cfu).

A number of prior studies have demonstrated that parenteral vaccination, when adjuvanted with ADP-ribosylating enterotoxins, including the modified double (R192G/L211A) mutant version of *E. coli* heat-labile toxin (dmLT) (62), can direct antigen-specific immune responses in the intestine (63–65). To explore the utility of parenteral vaccination with EtpA, we first questioned whether IM vaccination of rEtpA adjuvanted with dmLT would engender EtpA-specific mucosal antibody responses. Intramuscular vaccination of mice resulted in fecal IgA and IgG responses to EtpA, with fecal IgG being predominant (Fig. 1A). Notably, both IgA and IgG mucosal responses increased significantly following ETEC infection of parenterally immunized mice relative to PBS controls, where no response to infection alone was observed. These data suggested that parenteral vaccination with rEtpA, at least when adjuvanted with dmLT, can elicit mucosal antibody responses and may prime mucosal responses to this antigen elicited by ETEC infection.

### Comparison of dmLT to alum

We next compared serum and fecal responses to EtpA following parenteral vaccination with dmLT as the adjuvant, relative to vaccination with rEtpA adjuvanted with alum. Vaccination with either adjuvant preparation resulted in modest serum IgA responses but robust IgG responses (Fig. 1B). We observed modest increases in fecal IgA following H10407 challenge in mice immunized with dmLT/rEtpA, but not in mice where alum was used as the adjuvant. Anti-EtpA fecal IgG levels were increased in both groups, although somewhat higher in mice immunized with the dmLT-EtpA combination.

### The timing of rEtpA parenteral vaccination adjuvanted with dmLT dictates the outcome

**Fig 2:**
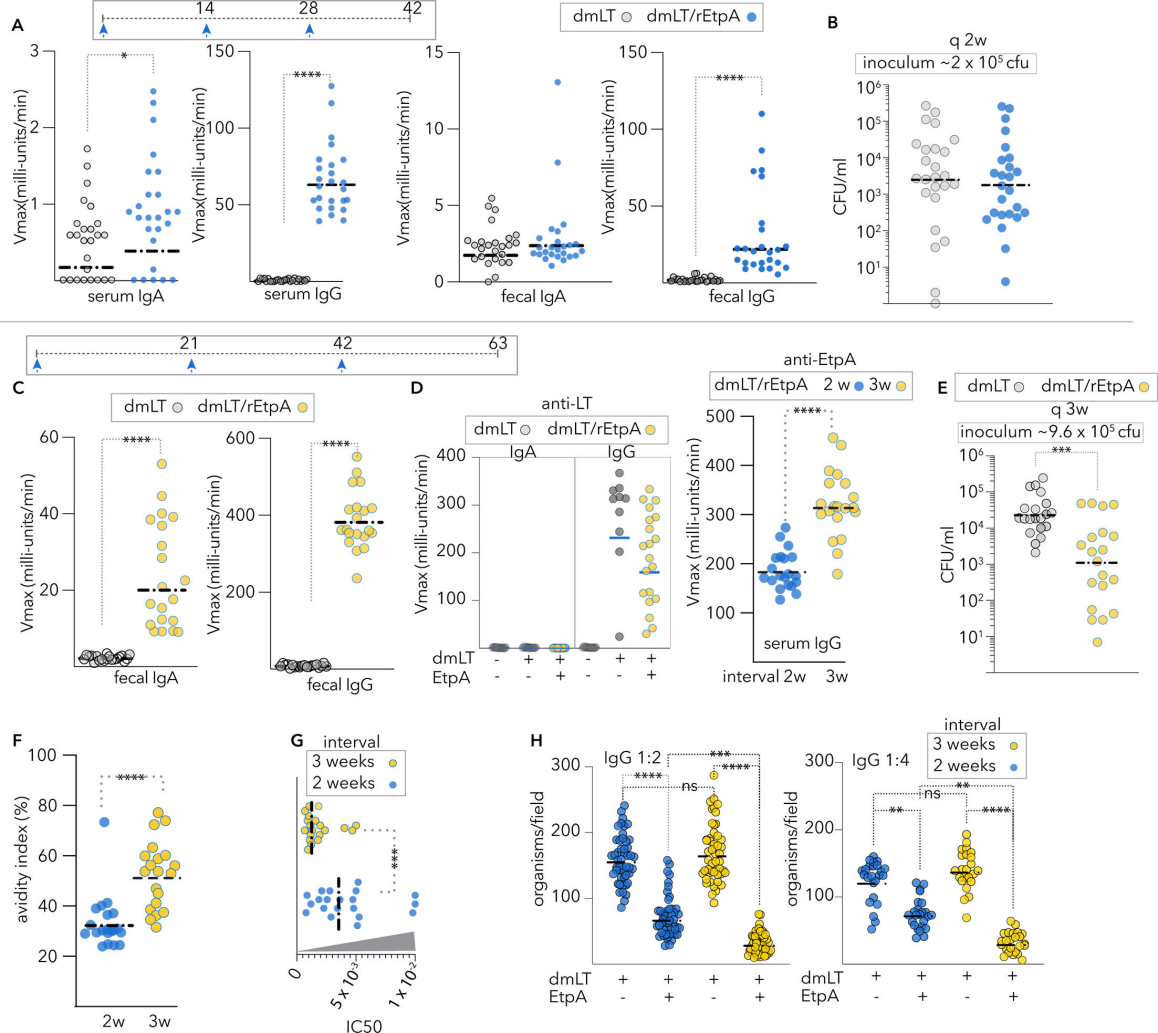
Vaccination timing impacts immunogenicity and protection. (**A**) Serum and fecal IgA and IgG values following IM immunization on days 0, 14, and 28 with rEtpA adjuvanted with dmLT. (Serum IgG measured at 1:100,000 dilution). (**B**) Intestinal colonization at day 42 in unvaccinated controls vs. mice vaccinated with rEtpA adjuvanted with dmLT at two-week intervals. (**C**) Anti-EtpA fecal IgA and IgA following IM vaccination at three-week intervals with dmLT/rEtpA vs. unvaccinated controls anti-EtpA fecal IgG following IM vaccination at three-week intervals with dmLT/rEtpA vs. unvaccinated controls. (**D**) (Left) Anti-LT serologic responses following vaccination with adjuvant only and EtpA adjuvanted with dmLT. (Right) Comparison of anti-EtpA serum IgG values (kinetic ELISA) of mice vaccinated with rEtpA/dmLT at two- or three-week intervals (sera diluted 1:50,000). (**E**) Intestinal colonization in unvaccinated controls vs. mice vaccinated with rEtpA adjuvanted with dmLT at three-week intervals. (**F**) Avidity of IgG antibody following vaccination with dmLT/rEtpA at two- or three-week intervals. (**G**) Vaccination at three-week intervals enhances the neutralization activity of IgG antibodies in rEtpA-A blood group binding assays. (**H**) IgG from mice vaccinated with dmLT/rEtpA at three-week intervals more effectively impairs ETEC adhesion to target HT29 cells. Dashed horizontal bars throughout represent geometric mean values. Statistical comparisons between groups by Kruskal–Wallis analysis. ****<0.0001, ***<0.001,*<0.05.

Next, we examined the protection afforded against ETEC small intestinal colonization by parenteral immunization in a murine infection model (49, 60). Mice were vaccinated IM with 10 µg of rEtpA adjuvanted with 1 µg of dmLT on days 0, 14, and 28 and then challenged on day 44 with ~2 × 10^5^ colony-forming units of jf876 to assess protection against ETEC colonization of the small intestine. We again observed IgG-predominant responses in both feces and sera (Fig. 2A). However, this was not associated with significant protection against intestinal colonization relative to adjuvant-only vaccinated controls (Fig. 2B).

Because the pace of antigen delivery can influence B cell maturation and antibody affinity, as well as the diversity of epitopes recognized (66, 67), we vaccinated a second group of animals with the same adjuvant and dose of rEtpA, but at three-week intervals (days 0, 21, and 42, followed by a challenge on day 63). Again, we observed a predominant fecal anti-EtpA IgG response to vaccination (Fig. 2C). Interestingly, mice vaccinated with rEtpA adjuvanted with dmLT on this more protracted schedule generally exhibited stronger serum IgG responses than those vaccinated on a two-week schedule (Fig. 2D) and were protected against intestinal colonization with ETEC relative to adjuvant-only controls (Fig. 2E). Finally, the avidity of serum antibodies was appreciably higher (Fig. 2F) following vaccination at three-week intervals, and these antibodies were significantly more effective in preventing EtpA interaction with target A blood group molecules (Fig. 2G) and EtpA-expressing ETEC adhesion to target A blood group + HT29 cells (Fig. 2H).

**Fig 3:**
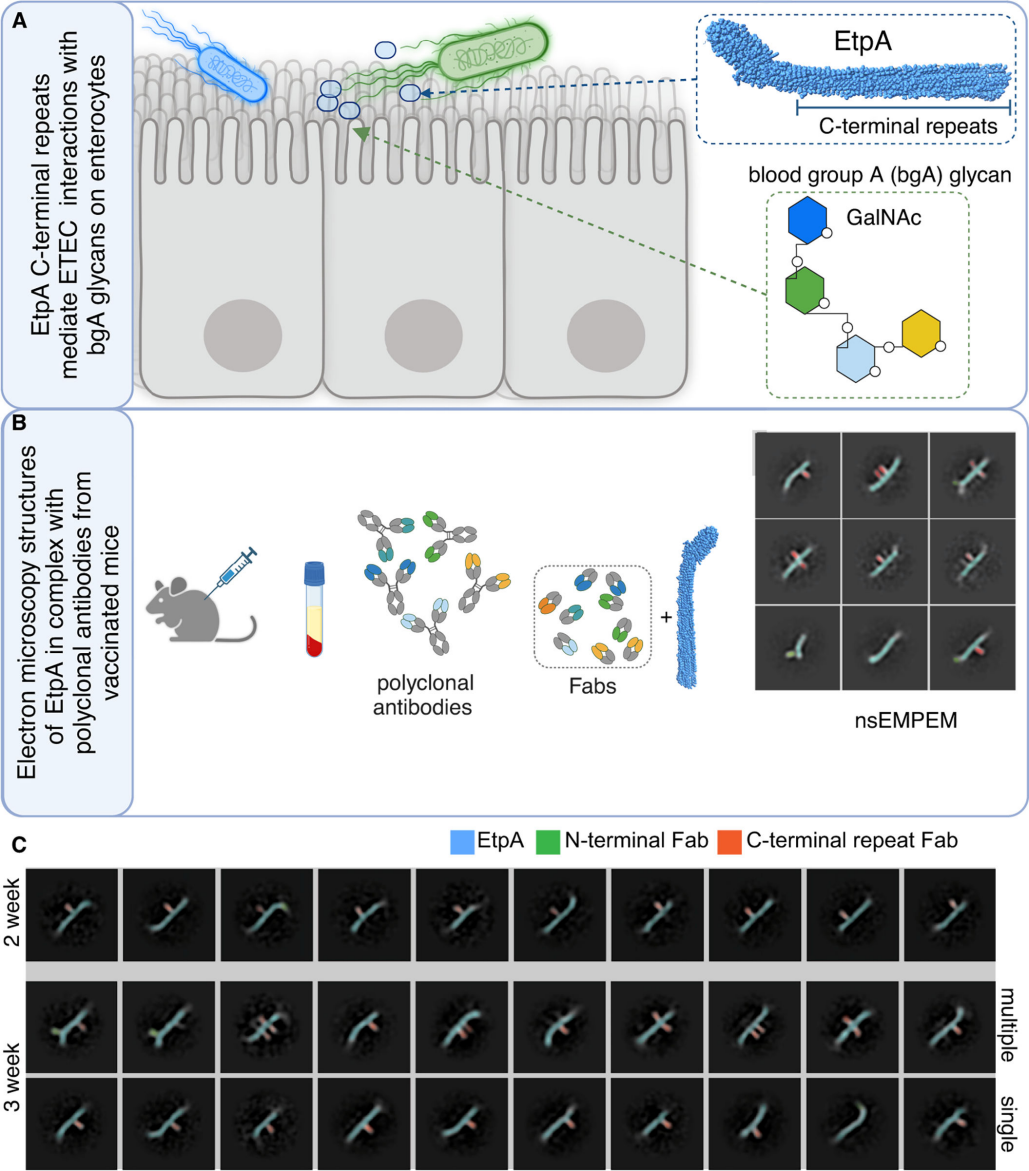
Vaccination administration schedule impacts antibody diversity. (**A**) Schematic of EtpA lectin activity wherein the C-terminal repeat region of EtpA (CTR) directs binding to A blood group glycans expressed on the surface of enterocytes. (**B**) Negative stain electron microscopy polyclonal antibody epitope mapping (nsEMPEM) protocol. Created in BioRender. Fleckenstein, J. (2025) https://BioRender.com/ee1smdr (**C**) nsEMPEM of IgG Fabs generated from sera of mice vaccinated every two weeks or every three weeks with rEtpA adjuvanted with dmLT. Selected two-dimensional class averages of rEtpA in complex with Fabs generated from sera of mice vaccinated every two weeks (top row). The middle panel shows multiple-Fabs/rEtpA molecules, and the bottom row shows single-Fabs/rEtpA from mice vaccinated every three weeks with rEtpA/dmLT. Pseudo-coloring reflects rEtpA (blue), CTR-binding Fabs (orange), and NTD-binding Fabs (green).

Our recent studies have shown that the C-terminal repeat region of EtpA is essential for its activity as a blood group A lectin and that monoclonal antibodies recognizing the repeat regions of EtpA interrupt binding to target glycans (Fig. 3A) (48). Therefore, to further characterize humoral responses to rEtpA vaccination, we performed negative stain electron microscopy-based polyclonal epitope mapping (nsEMPEM) (68) on Fab fragments generated from IgG isolated from mice vaccinated with rEtpA adjuvanted with dmLT (Fig. 3B). This resulted in 1,575 single Fab-EtpA complexes observed in the two-week sample compared to 1,527 single Fab and 1,338 multi-Fab complexes in the three-week sample (Fig. S1). Qualitatively, we observed more diverse 2D class averages of Fab-EtpA complexes in the three-week samples, including classes where rEtpA was complexed with multiple (two and sometimes three) Fabs (Fig. 3C), including multiple Fabs targeting neutralizing epitopes on the C-terminal repeat domain (CTR), which allow for binding avidity of intact IgG (48). Collectively, these results demonstrate that immunization timing can profoundly affect both the quantity and quality of polyclonal antibodies elicited against rEtpA, findings that coincide with the ability to neutralize EtpA lectin activity *in vitro* and, ultimately, protection against colonization *in vivo*.

### Parenteral vaccination with alum-adjuvanted rEtpA impairs ETEC colonization

**Fig 4:**
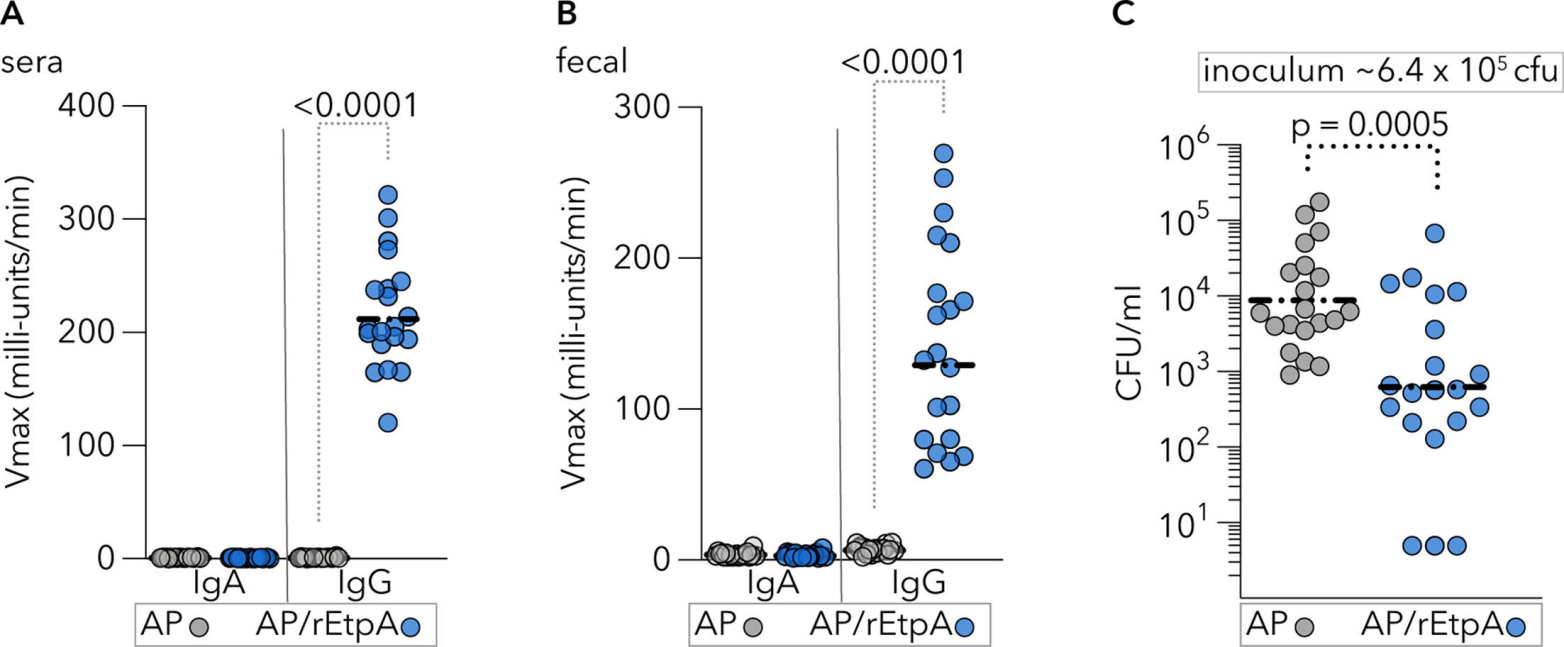
Intramuscular vaccination with alum-adjuvanted EtpA protects against intestinal colonization with ETEC. CD-1 mice (*n* = 20/group) were vaccinated (days 0, 21, and 42) with Adju-Phos (AP) alone or EtpA adjuvanted with AP. (**A**) Serum IgA and IgG levels following vaccination with AP alone or rEtpA adjuvanted with AP (sera obtained from terminal bleed at day 63). (**B**) Anti-EtpA fecal antibody levels following vaccination. Fecal antibodies were measured in suspensions of fecal pellets obtained on day 62 prior to the challenge. (**C**) Intestinal colonization of mice following a challenge on day 63. Comparisons by Mann-Whitney (2-tailed). Dashed horizontal lines represent geometric mean values.

Although mutant versions of heat-labile toxin have been used in multiple clinical trials, including recent subunit ETEC vaccine studies (69), many vaccines licensed over the past several decades have been adjuvanted with alum (70). Therefore, to extend our observations, we examined immune responses and protection afforded by vaccination with rEtpA vaccinated with aluminum phosphate, following the more protracted vaccination schedule on days 0, 21, and 42, with a challenge on day 63. Alum-adjuvanted IM vaccination with rEtpA yielded a predominant IgG response in both serum (Fig. 4A) and stool (Fig. 4B). Despite little discernible IgA response, vaccinated mice exhibited reduced levels of intestinal colonization with ETEC following challenge (Fig. 4C), further suggesting that rEtpA could afford protection as part of a parenterally delivered subunit vaccine.

## DISCUSSION

Since their discovery as a cause of acute cholera-like watery diarrhea more than 50 years ago (71, 72), enterotoxigenic *E. coli* have presented formidable challenges to vaccine development. In contrast to *Vibrio cholerae*, which have largely been limited to only a few serotypes, the plasmid-encoded virulence genes of ETEC are present in a genetically diverse group of *E. coli* comprised of a multitude of O- and H-serogroups (73).

The main targets for vaccine development over the past five decades have been plasmid-encoded antigens known as colonization factors (CFs) (74). Early enthusiasm for CF antigens was in part stimulated by studies demonstrating that a strain (H10407-P), which had been cured of a plasmid-carrying gene for the CFA/I colonization factor, was effectively avirulent relative to the ETEC wild-type H10407 strain (75). Subsequent studies demonstrated that passive immunization with hyperimmune bovine milk immunoglobulin, raised against CFA/I, afforded significant protection against challenge with ETEC H10407 (76), engendering additional support for CF-based ETEC vaccine development. More recently, sophisticated structural studies have facilitated the development of recombinant tip adhesin molecules, which likewise offer protection in passive (77) and active (78) immunization studies.

Despite these advances, the development of a broadly protective subunit vaccine for ETEC based exclusively on CFs will present significant challenges given the extraordinary diversity of these antigens described to date. Notably, the large mosaic plasmid encoding CFA/I, which was previously cured from H10407, was later shown to encode the etpBAC two-partner secretion locus responsible for the production of the EtpA glycoprotein (39), as well as the EatA mucinase autotransporter (79).

It is likely that subunit vaccine development incorporating EtpA will require further exploration and optimization of adjuvants, as has been done with fimbrial subunits (80). Nevertheless, the present studies suggest that parenteral immunization with EtpA is feasible and that this antigen could expand coverage afforded by current CF-centered approaches.

Recent studies have demonstrated that non-mucosal vaccination adjuvanted with dmLT can elicit the antigen-specific migration of CD4+ antigen-specific T cells (63) and B cells into intestinal mucosa, resulting in fecal IgA (81). In the current study, IM vaccination with EtpA adjuvanted by dmLT elicited modest fecal IgA and IgG responses, with demonstrable increases in antigen-specific antibody responses following infection. IgA produced at mucosal sites is thought to exclude enteric pathogens, in part by clumping the growing bacteria to accelerate their clearance from the intestine (82).

The majority of parenteral vaccines, including several in the Expanded Program on Immunization, have been adjuvanted with alum (83). Despite a traditional focus on mucosal protection mediated by IgA, IgG has been shown to play a complementary, if not dominant, role in protecting against some intestinal pathogens (84), in part by targeting surface virulence factors and triggering their elimination by transmigrating neutrophils (85). The studies reported here suggest that the fecal IgG responses generated by immunization with recombinant EtpA, adjuvanted with either dmLT or alum, may sufficiently target this surface-expressed adhesin to provide protection. The present studies suggest that both the quality and quantity of antibodies produced may be dependent on vaccination strategy. Nevertheless, serum antibodies may not provide an accurate proxy for antibodies that are delivered to mucosal surfaces.

The ability to define precise mechanistic correlates of protection remains a challenge for many vaccines targeting mucosal pathogens (86–88). However, a detailed understanding of antigenic structure–function relationships can aid in characterizing neutralizing antibody responses that lead to protection. The recent determination of the EtpA structure and the elucidation of the essential role played by its C-terminal repeat domain in directing interactions with target glycans on mucosal epithelia (48) can facilitate the development of assays that help to define its role as a protective subunit antigen and aid the identification of correlates of protection.

## SUPPLEMENTAL MATERIAL

Fig. S1 iai.00601-24-s0001.pdf Negative stain EMPEM strategy.

ASM does not own the copyrights to Supplemental Material that may be linked to, or accessed through, an article. The authors have granted ASM a non-exclusive, world-wide license to publish the Supplemental Material files. Please contact the corresponding author directly for reuse.
