# Supplementary material for: Parenteral vaccination with recombinant EtpA glycoprotein impairs enterotoxigenic E. coli colonization
Source: Infect Immun. 2025 May 1;93(6):e00601-24. doi: 10.1128/iai.00601-24 (PMC12150687; doi:10.1128/iai.00601-24)
Supplement: Fig. S1 — Negative stain EMPEM strategy. [file iai.00601-24-s0001.pdf]

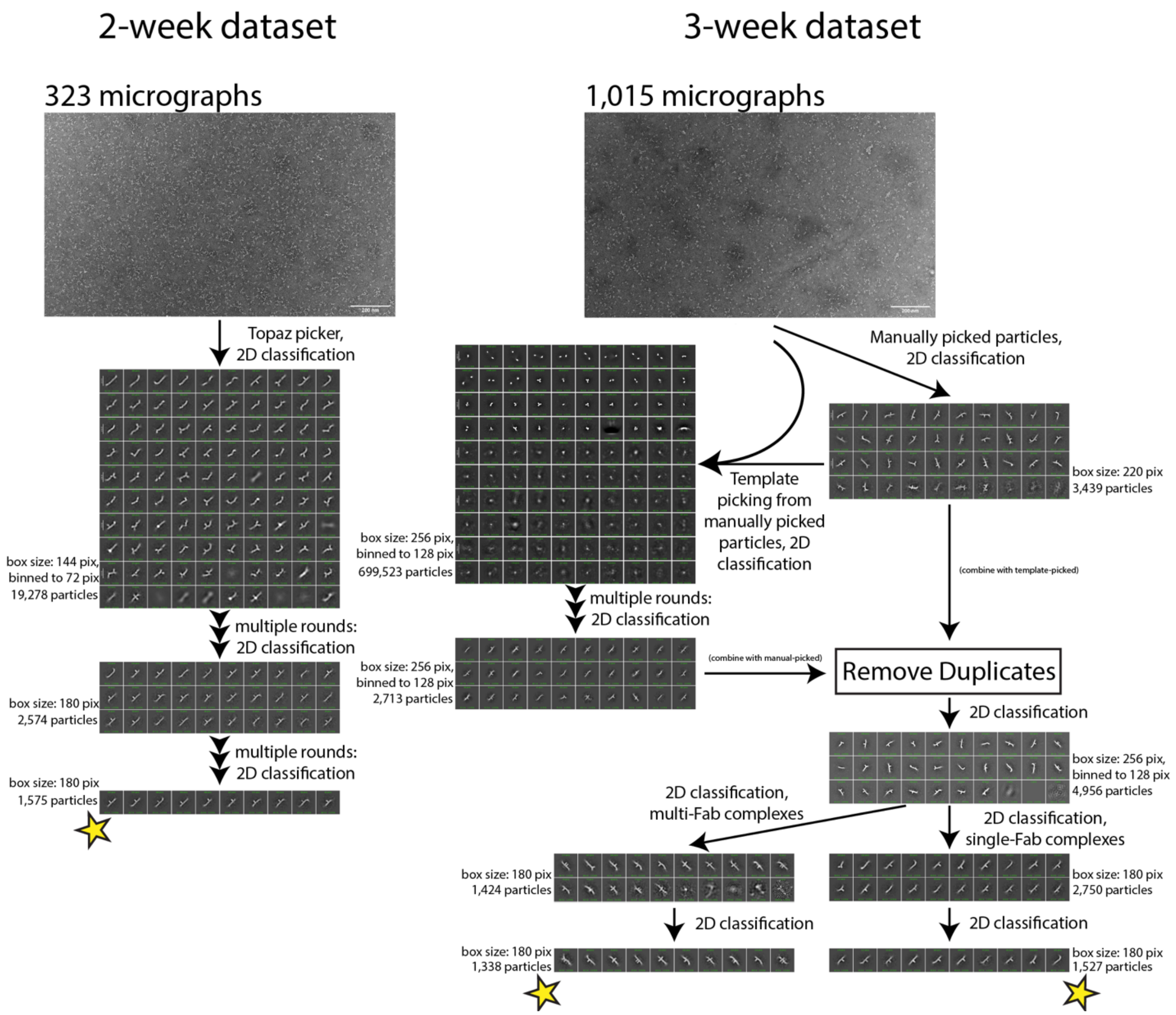


**Supplementary figure 1. nsEMPEM particle identification strategy.**
323 micrographs generated in the Washington University Center for Cellular Imaging (WUCCI) were used to identify classes in the 2-week dataset resulting in a total of 1575 2D negative stain EMPEM (nsEMPEM) complexes after data refinement. 1,015 micrographs were processed for the 3-week samples resulting in two separate 2 D classifications containing either multi-Fab complexes (1,338 particles) or single-Fab complexes (1,527 particles) after duplicate filtering and data refinement.
